# Nerandomilast in Autoimmune-Associated Interstitial Lung Diseases: Translating Evidence from Progressive Pulmonary Fibrosis Studies

**DOI:** 10.3390/jcm15062166

**Published:** 2026-03-12

**Authors:** Fabio Perrotta, Domenica Francesca Mariniello, Giulia M. Stella, Raffaella Pagliaro, Filippo Scialò, Vasiliki Liakouli, Giulio Forte, Francesco Ciccia, Andrea Bianco, Vito D’Agnano

**Affiliations:** 1 Department of Translational Medical Sciences, University of Campania Luigi Vanvitelli, Via Leonardo Bianchi, 80131 Naples, Italy; raffaella.pagliaro@studenti.unicampania.it (R.P.);; 2 Clinic of Respiratory Diseases “Luigi Vanvitelli”, A.O. dei Colli, Monaldi Hospital, 80131 Naples, Italy; 3 Department of Internal Medicine and Medical Therapeutics, Medical School, University of Pavia, 27100 Pavia, Italy; 4 CEINGE-Biotecnologie Avanzate Franco Salvatore, 80145 Naples, Italy; 5 Dipartimento di Medicina Molecolare e Biotecnologie Mediche, Università degli Studi di Napoli Federico II, 80131 Naples, Italy; 6 Rheumatology Unit, Department of Precision Medicine, University of Campania Luigi Vanvitelli, 80131 Naples, Italy

**Keywords:** nerandomilast, PDE4B inhibition, progressive pulmonary fibrosis, SARD-ILD, antifibrotic therapy

## Abstract

Systemic autoimmune rheumatic disease-associated interstitial lung disease (SARD-ILD) comprises a heterogeneous group of fibrosing lung disorders frequently complicated by progressive pulmonary fibrosis, a phenotype associated with accelerated lung function decline and increased mortality. Although antifibrotic therapies have improved clinical outcomes, significant unmet needs remain, particularly regarding treatment tolerability and integration with background immunosuppressive strategies. Preferential phosphodiesterase-4B (PDE4B) inhibition has emerged as a novel therapeutic approach targeting both inflammatory and fibrotic pathways through modulation of intracellular cyclic adenosine monophosphate signaling. This narrative review summarizes the biological rationale and emerging clinical evidence supporting nerandomilast, an oral preferential PDE4B inhibitor, in autoimmune-associated interstitial lung diseases. Preclinical data indicate that PDE4B inhibition may attenuate fibroblast activation, inflammatory signaling, and extracellular matrix deposition. Clinical trials conducted in progressive pulmonary fibrosis populations have demonstrated a reduction in lung function decline, with subgroup analyses suggesting potential benefit in autoimmune-related diseases, although evidence remains limited. The safety profile appears mainly characterized by gastrointestinal adverse events, with ongoing evaluation of neuropsychiatric safety and drug interactions in complex autoimmune populations. Overall, nerandomilast represents a promising investigational strategy bridging antifibrotic and immunomodulatory mechanisms, warranting further dedicated studies in SARD-ILD.

## 1. Introduction

Systemic autoimmune rheumatic disease-associated interstitial lung disease (SARD-ILD) encompasses a heterogeneous group of fibrosing lung disorders occurring in the context of systemic autoimmunity, including rheumatoid arthritis, systemic sclerosis and idiopathic inflammatory myopathies. Epidemiological studies indicate that ILD affects a substantial proportion of patients across SARDs, with reported prevalence ranging from approximately 47% in systemic sclerosis and 41% in idiopathic inflammatory myopathies to 11–17% in rheumatoid arthritis and Sjögren’s disease, respectively. Overall, SARD-ILD represents a major determinant of morbidity and mortality, contributing significantly to healthcare utilization and long-term disease burden [1]. Beyond its heterogeneous prevalence, SARD-ILD displays a highly variable clinical trajectory. While some patients experience stable inflammatory disease, a considerable subset develops a progressive fibrosing phenotype characterized by worsening respiratory symptoms, decline in lung function and increasing fibrotic abnormalities on high-resolution computed tomography. Recent estimates suggest that up to 40–50% of patients with autoimmune-associated ILD may develop progressive pulmonary fibrosis (PPF), a disease behavior associated with markedly reduced survival compared with non-progressive disease, with median survival decreasing from approximately 8–10 years to nearly 4 years in some cohorts [2]. The recognition of progressive fibrosing behavior across non-idiopathic pulmonary fibrosis interstitial lung diseases has led to a paradigm shift in disease classification. The 2022 ATS/ERS/JRS/ALAT clinical practice guideline introduced a standardized definition of progressive pulmonary fibrosis based on a combination of symptomatic worsening, physiological decline and radiological progression occurring within a defined time frame. This framework has highlighted that only a subset of fibrosing ILDs progresses despite initial disease-specific management, emphasizing the need for targeted therapeutic strategies in patients with ongoing fibrotic activity [3]. The recognition of progressive pulmonary fibrosis (PPF) as a shared disease behavior has fundamentally reshaped therapeutic paradigms by shifting management beyond purely immunosuppressive strategies toward direct targeting of fibrogenesis. In randomized clinical trials, antifibrotic therapy (pirfenidone and nintedanib) demonstrated a clinically meaningful reduction in the rate of lung function decline (Table 1). In particular, data from INBUILD and SENSCIS trials confirmed that targeting fibrotic pathways may modify disease progression in immune-mediated ILD. However, despite these advances, important unmet needs remain regarding tolerability and integration with immunosuppressive therapy [4,5]. Beyond randomized trials, emerging registry-based evidence has reinforced these findings under real-world conditions. In a longitudinal multicenter study from the NEREA registry including patients with autoimmune-related ILD who developed progressive pulmonary fibrosis, functional respiratory impairment occurred at a high incidence rate of 57.4 events per 100 patient-years, with nearly 69% of patients experiencing functional deterioration within 24 months after PPF onset, underscoring the aggressive natural history of this phenotype [6]. Importantly, after multivariable adjustment, antifibrotic therapy significantly reduced the risk of lung function worsening, with hazard ratios of 0.58 (95% CI 0.39–0.85) for nintedanib and 0.68 (95% CI 0.50–0.94) for pirfenidone compared with no antifibrotic therapy [6]. However, important unmet needs remain. A proportion of patients continues to exhibit disease progression despite antifibrotic therapy, treatment-limiting adverse events frequently affect tolerability, and the integration of antifibrotics with background immunosuppressive regimens raises complex safety and drug-interaction considerations in routine clinical practice [2,7]. In a retrospective cohort comparing antifibrotic tolerability between SARD and non-SARD ILD, the cumulative incidence of treatment discontinuation due to adverse events did not significantly differ across groups; however, important differences emerged in treatment exposure and adverse-event profiles. Notably, among patients receiving nintedanib, the median duration of therapy prior to adverse-event-related discontinuation was markedly shorter in the SARD-ILD group compared with non-SARD ILD [8]. In this evolving therapeutic landscape, nerandomilast—a preferential phosphodiesterase-4B inhibitor with both anti-fibrotic and immunomodulatory properties—has emerged as a promising investigational therapy. By targeting intracellular cyclic adenosine monophosphate signaling and modulating key inflammatory and fibrogenic pathways, nerandomilast may offer a novel pharmacological approach bridging antifibrotic efficacy with immune regulation. As clinical development increasingly relies on the progressive pulmonary fibrosis paradigm, evidence derived from progressive pulmonary fibrosis studies provides a framework to explore the potential role of nerandomilast in patients with SARD-ILD [9]. Therefore, this narrative review aims to summarize the biological rationale and emerging clinical evidence supporting preferential PDE4B inhibition with nerandomilast in autoimmune-associated interstitial lung diseases, with particular attention to data derived from PPF trials.

## 2. Methods

### Literature Search Strategy and Study Selection

A structured literature search was conducted in PubMed/MEDLINE and Scopus since January 2010 to January 2026. The following keyword combinations were used: “nerandomilast” OR “BI 1015550” OR “PDE4B inhibitor” OR “phosphodiesterase-4 inhibition” AND “interstitial lung disease” OR “progressive pulmonary fibrosis” OR “autoimmune interstitial lung disease” OR “connective tissue disease-associated ILD” OR “SARD-ILD” OR “systemic sclerosis-associated ILD” OR “rheumatoid arthritis ILD” OR “idiopathic pulmonary fibrosis”. Additional searches included terms focused on safety and tolerability, such as “adverse events”, “safety profile”, “tolerability”, “diarrhea”, “psychiatric safety”, and “suicidal ideation”, as well as combinations including “nintedanib”, “pirfenidone”, and “antifibrotic therapy” to contextualize comparative safety data. Randomized clinical trials, translational studies, mechanistic investigations, registry analyses, and relevant narrative reviews were considered for inclusion if they provided data relevant to PDE4 inhibition, progressive fibrosing ILD, or autoimmune-associated ILD. Given the limited number of studies specifically addressing SARD-ILD, evidence from idiopathic pulmonary fibrosis trials was also included when relevant to safety or pharmacologic rationale, which is explicitly indicated in the text. Although a systematic search strategy was applied to identify the available literature, the present manuscript is structured as a narrative review, aiming to provide a critical and clinically oriented interpretation of emerging data rather than a formal meta-analysis or quantitative systematic review.

## 3. Biological Rationale: PDE4 Inhibition and Fibrogenesis

Pulmonary fibrosis is sustained by an interplay between epithelial injury, innate/adaptive immune activation, and fibroblast-driven extracellular matrix (ECM) deposition [16,17,18,19]. Cyclic adenosine monophosphate (cAMP) is a central intracellular second messenger that broadly constrains inflammatory activation and modulates profibrotic cellular programs. A key feature of cAMP biology is its compartmentalized signaling, whereby localized pools of cAMP govern discrete downstream effects; phosphodiesterases (PDEs) provide spatial and temporal “brakes” by degrading cAMP in specific microdomains. In this framework, PDE4 enzymes—cAMP-specific phosphodiesterases—act as critical regulators of cAMP-dependent pathways, shaping cell-specific responses relevant to fibrosis and inflammation [20,21].

### PDE4 Inhibition as an Antifibrotic Strategy: Fibroblast-, Immune-, and Epithelium-Relevant Effects

Multiple lines of preclinical evidence support PDE4 inhibition as a strategy with dual anti-inflammatory and antifibrotic potential. In fibroblast-centric systems, PDE4 inhibition has been associated with attenuation of transforming growth factor-β (TGF-β)-driven profibrotic phenotypes, including reduced fibroblast-to-myofibroblast differentiation and suppression of contractile and migratory behaviors in relevant in vitro models (e.g., collagen gel contraction and chemotaxis) [22].

These cellular effects are biologically coherent with the concept that preservation of intracellular cAMP can amplify residual endogenous antifibrotic mediators (including prostaglandin E2-related pathways) and restrain myofibroblast programs [22]. The antifibrotic signal extends to in vivo models. In a murine model of fibrosis driven by targeted type II alveolar epithelial cell (AEC) injury, a setting characterized by relatively modest inflammation and diffuse fibrosis, PDE4 inhibitors (including roflumilast, piclamilast, and a novel compound) reduced fibrosis using both prophylactic and therapeutic regimens, with efficacy reported as comparable to pirfenidone and nintedanib in that experimental system; protection was also associated with reduced weight loss and lower plasma surfactant protein-D (SP-D), consistent with an epithelial-protective signal [22]. Complementary evidence comes from bleomycin-induced pulmonary fibrosis, where selective PDE4 inhibition (cilomilast) reduced early alveolar inflammatory cell burden in bronchoalveolar lavage (especially macrophages and lymphocytes), modulated inflammatory cytokine expression (including reduced TNF-α mRNA), improved later physiological parameters (e.g., compliance), attenuated histological fibrosis, and showed trends toward reduced collagen content and improved survival [23]. A practical barrier to “classic” pan-PDE4 inhibition is tolerability, particularly gastrointestinal adverse events that have limited chronic use of some agents. This has increased interest in preferential PDE4B inhibition, aiming to retain anti-inflammatory/antifibrotic activity while potentially reducing emesis-related liability attributed largely to PDE4D inhibition [24].

The preferential targeting of PDE4B is particularly attractive in fibrosing ILD, given its expression in both immune and structural lung cells involved in fibrogenesis [25].

**Figure 1:**
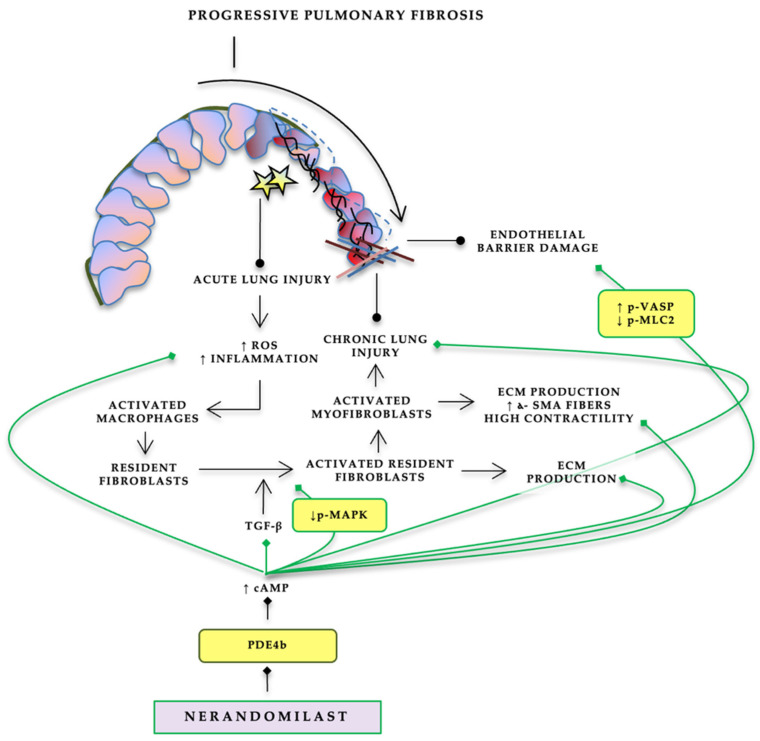
Proposed mechanism of action of preferential PDE4B inhibition in progressive pulmonary fibrosis. Persistent injury (stars) drives fibroblast activation and differentiation into contractile myofibroblasts under the influence of transforming growth factor-β (TGF-β), resulting in extracellular matrix (ECM) deposition and progressive architectural distortion. Phosphodiesterase-4 (PDE4) regulates intracellular cyclic adenosine monophosphate (cAMP) signaling, a key modulator of inflammatory and profibrotic pathways. Preferential PDE4B inhibition with nerandomilast is hypothesized to increase intracellular cAMP levels, thereby attenuating inflammatory activation, limiting fibroblast-to-myofibroblast transition, and reducing ECM production, ultimately slowing fibrotic progression. Roxygen species (ROS) production and inflammatory signaling are over-expressed during acute damage—i.e., viral infection—leading to macrophage activation and stimulation of resident fibroblasts. Abbreviations: PDE4, phosphodiesterase-4; PDE4B, phosphodiesterase-4B; cAMP, cyclic adenosine monophosphate; ROS, reactive oxygen species; TGF-β, transforming growth factor-beta; ECM, extracellular matrix; α-SMA, alpha-smooth muscle actin.

Nerandomilast (BI 1015550) is an orally administered preferential PDE4B inhibitor with reported selectivity favoring PDE4B over PDE4D (approximately nine-fold in human enzymes, as summarized in contemporary reviews) [26]. Preclinical observations summarized in recent translational reviews describe anti-inflammatory activity (e.g., suppression of LPS-induced TNF-α in murine whole blood ex vivo; inhibition of LPS-driven neutrophil influx into bronchoalveolar lavage (BAL) in a shrew model), as well as antifibrotic activity including inhibition of primary lung fibroblast proliferation, attenuation of TGF-β1-induced myofibroblast transformation, and downregulation of ECM gene expression programs (Figure 1). In fibrotic lung models, nerandomilast has also been associated with reductions in inflammatory cell populations in BAL and modulation of profibrotic pathways at transcript/protein levels, supporting a mechanistic profile that spans both inflammatory and fibrotic axes relevant to progressive pulmonary fibrosis [27].

Compartmentalized cAMP signaling biology along with convergent preclinical data provide a coherent rationale for PDE4 inhibition—and specifically PDE4B-preferential inhibition—as a candidate approach for progressive fibrosing ILD. This dual-pathway concept is particularly attractive in SARD-ILD, where immune-mediated injury and fibrotic remodeling coexist and where progression may occur despite immunosuppression and/or antifibrotic therapy, motivating evaluation of agents capable of modulating both components of disease biology.

## 4. Nerandomilast Clinical Evidence in Progressive SARD-ILD: Insights from the FIBRONEER-ILD Trial

The FIBRONEER-ILD phase III trial [15] evaluated the efficacy of the preferential phosphodiesterase-4B inhibitor nerandomilast in patients with PPF across a heterogeneous spectrum of non-IPF fibrosing interstitial lung diseases. The study randomized patients in a 1:1:1 ratio to nerandomilast 9 mg twice daily, nerandomilast 18 mg twice daily, or placebo, with stratification according to background nintedanib use and high-resolution computed tomography (HRCT) pattern. The primary endpoint was the absolute change in FVC at week 52, reflecting the contemporary paradigm of targeting fibrotic progression independent of underlying ILD etiology. In the overall trial population, nerandomilast significantly reduced the rate of lung function decline compared with placebo. At week 52, the adjusted mean change in FVC was −165.8 mL in the placebo group, −84.6 mL in the nerandomilast 9 mg group, and −98.6 mL in the nerandomilast 18 mg group, corresponding to adjusted treatment differences versus placebo of +81.1 mL (95% CI, 46.0 to 116.3) and +67.2 mL (95% CI, 31.9 to 102.5), respectively. These effects were consistent across sensitivity analyses and prespecified subgroups, including patients receiving background nintedanib therapy. Beyond the primary endpoint, nerandomilast demonstrated a favorable trend across key clinical outcomes. The composite secondary endpoint of first acute exacerbation of ILD, respiratory-related hospitalization, or death occurred less frequently in the nerandomilast groups than in the placebo group, with hazard ratios of 0.77 (95% CI, 0.59–1.01) for the 18 mg dose and 0.88 (95% CI, 0.68–1.14) for the 9 mg dose. Mortality was numerically lower with nerandomilast, with hazard ratios for death of 0.48 (95% CI, 0.30–0.79) and 0.60 (95% CI, 0.38–0.95) for the 18 mg and 9 mg doses, respectively [15].

### 4.1. Proportion and Composition of Autoimmune Disease-Related ILD Within the Trial Population

Within this overall efficacy framework, a closer examination of the autoimmune ILD subgroup provides important insights into the potential role of nerandomilast in SARD-associated disease. Among the 1176 patients who received at least one dose of study drug, the prespecified subgroup of “autoimmune ILDs” accounted for approximately 27.6% of the total population, with balanced representation across treatment arms (25.5% in the placebo group, 28.5% in the nerandomilast 9 mg group, and 28.9% in the nerandomilast 18 mg group). This subgroup extended beyond the core and electronic case report forms (eCRF) diagnoses of rheumatoid arthritis-associated ILD (RA-ILD), systemic sclerosis-associated ILD (SSc-ILD), and mixed connective tissue disease-associated ILD (MCTD-ILD), as the statistical analysis plan allowed inclusion of additional autoimmune phenotypes originally categorized as “other fibrosing ILDs” but subsequently confirmed by steering committee review. Using a conservative definition limited to eCRF diagnoses alone, RA-ILD, SSc-ILD, and MCTD-ILD together represented approximately 20.4% of the total trial population, underscoring that a meaningful fraction of autoimmune disease-related ILD cases was embedded within broader diagnostic categories. This distinction is important for interpretation, as the clinical heterogeneity of autoimmune ILD may influence both baseline disease trajectory and responsiveness to antifibrotic therapy [15].

### 4.2. Effect of Nerandomilast on Lung Function Decline in the Autoimmune ILD Subgroup

Prespecified subgroup analyses demonstrated a numerically favorable treatment effect of nerandomilast in patients with autoimmune ILD. At week 52, the adjusted mean difference in change from baseline FVC compared with placebo was +45.9 mL (95% CI −20.8 to 112.6) for nerandomilast 9 mg twice daily and +42.2 mL (95% CI −24.9 to 109.3) for nerandomilast 18 mg twice daily. Although the direction of effect was consistent with the overall study population, confidence intervals crossed zero, reflecting limited precision and the absence of statistical power for autoimmune-specific inference. Notably, the magnitude of benefit observed in autoimmune ILDs appeared smaller than in the overall cohort, in which adjusted differences versus placebo reached approximately +81 mL and +67 mL for the 9 mg and 18 mg doses, respectively. The apparent attenuation of effect size in autoimmune ILD should be interpreted cautiously, as subgroup estimates were not powered for efficacy comparisons and displayed wide confidence intervals. These findings suggest that while nerandomilast may attenuate lung function decline in autoimmune-related PPF, more robust data are needed to confirm these trends [15].

### 4.3. Impact of Background Immunosuppression Restrictions on External Validity

A critical aspect influencing interpretation of the autoimmune ILD subgroup is the trial’s management of concomitant immunosuppressive therapy. While stable immunosuppressant use was permitted at enrollment, several agents commonly employed as disease-modifying therapies in SARD-ILD—including mycophenolate mofetil or sodium, rituximab, tocilizumab, and cyclophosphamide—were not allowed at baseline and could only be introduced after six months in the event of worsening systemic disease. Furthermore, changes in immunosuppressive therapy during the first six months were restricted, and prednisone doses exceeding 15 mg/day were not permitted at enrollment. Pirfenidone use was also prohibited. These protocol-mandated constraints likely resulted in an autoimmune ILD population that differs from real-world SARD-ILD cohorts, in which early optimization of systemic immunomodulatory therapy is often central to disease control and may independently influence FVC trajectories. Consequently, the observed treatment effect of nerandomilast in this subgroup must be interpreted within the context of a therapeutic environment where cornerstone immunosuppressive agents were partially withheld during a clinically relevant portion of follow-up. Additional immunosuppressive therapy was initiated in a small proportion of patients during the trial despite baseline restrictions. According to the supplementary analysis, mycophenolate mofetil was started in 2.0% of patients in the placebo and nerandomilast 9 mg groups and in 0.8% of those receiving nerandomilast 18 mg, while tocilizumab initiation occurred in 1.3% and 0.3% of patients in the nerandomilast 9 mg and 18 mg groups, respectively. Cyclophosphamide initiation was rare and observed only in the placebo arm (0.8%). Overall, initiation of immunosuppressive therapy during treatment remained uncommon, suggesting that the autoimmune ILD subgroup was largely managed in a relatively immunomodulation-restricted setting, which may have influenced the magnitude and interpretability of the antifibrotic treatment effect [15].

### 4.4. Safety and Tolerability

Preferential PDE4B inhibition has a safety profile that is biologically consistent with modulation of cyclic adenosine monophosphate signaling and with prior experience from the broader PDE4 inhibitor class. Mechanistically, gastrointestinal toxicity—particularly diarrhea—likely reflects enhanced epithelial secretion and altered intestinal motility driven by increased intracellular cAMP levels, whereas neuropsychiatric monitoring remains relevant given historical signals associated with non-selective PDE4 inhibitors [15]. Clinical safety data derive from both idiopathic pulmonary fibrosis (IPF) and PPF trials. In the phase 2 randomized study conducted in patients with IPF, adverse events occurred in 65% versus 52% of patients receiving nerandomilast versus placebo in the absence of background antifibrotic therapy and in 73% versus 68% among those receiving background antifibrotics. Diarrhea was reported in 17% versus 8% without background antifibrotics and 31% versus 16% with background therapy, representing the most frequent adverse event. Discontinuations due to adverse events occurred only in the active treatment groups (6% without background antifibrotics and 20% with background antifibrotics), while serious adverse events were reported in 6% versus 20% without background antifibrotics and 6% versus 0% with background therapy. Importantly, no on-treatment events related to depression or suicidal behavior were reported during active treatment, although a single episode of suicidal ideation type 1 was documented after study completion [28]. In the phase 3 FIBRONEER-ILD trial, diarrhea remained the most frequently reported adverse event, occurring in 36.6% of patients receiving nerandomilast 18 mg, 29.5% receiving 9 mg, and 24.7% receiving placebo. Permanent treatment discontinuation due to adverse events occurred in 10.0%, 8.1%, and 10.2% of patients in the 18 mg, 9 mg, and placebo groups, respectively, with diarrhea leading to discontinuation in 2.6%, 1.3%, and 0.5%. Rates of serious adverse events and adverse events of special interest—including vasculitis, depression, or suicidality—were balanced across treatment arms [15]. Interpretation of psychiatric safety requires consideration of trial-specific eligibility criteria. In FIBRONEER-ILD, patients with acute or severe depression defined by a Hospital Anxiety and Depression Scale (HADS) subscore greater than 14 were excluded, as were individuals with suicidal behavior within the previous two years or suicidal ideation type 4 or 5 according to the Columbia–Suicide Severity Rating Scale (C-SSRS). Prospective monitoring with HADS and C-SSRS was mandated throughout the study, and emergence of HADS scores above 14 or severe suicidal ideation constituted withdrawal criteria [15]. Finally, extrapolation of safety findings to SARD-ILD populations must consider limitations in available interaction data with immunosuppressive therapies. Several cornerstone immunomodulatory agents—including mycophenolate, rituximab, tocilizumab, and cyclophosphamide—were not permitted at enrollment, and modifications to immunosuppressive regimens were restricted during the first six months of the trial. As previously reported, the initiation of new immunosuppressive therapy during treatment was uncommon, with mycophenolate introduced in 2.0%, 2.0%, and 0.8% of patients and tocilizumab in 0%, 1.3%, and 0.3% of patients across placebo, 9 mg, and 18 mg groups, respectively. These low exposure rates limit conclusions regarding drug–drug interactions, infection risk under combination therapy, and real-world tolerability in the polypharmacy typical of systemic autoimmune disease [15,28].

### 4.5. Ongoing Clinical Trial

One phase 3 RCT (NCT06806592) is ongoing to evaluate the Efficacy and Safety of Nerandomilast Over at Least 26 Weeks in Patients With SARD-ILD. Participants continue immunosuppressant treatment for their underlying rheumatic disease. The primary outcome is absolute change from baseline in quantitative interstitial lung disease (QILD) score [%] on HRCT at week 26. The Secondary Outcome Measures are absolute change from baseline in quantitative lung fibrosis (QLF) score [%], in quantitative ground glass opacity (QGGO) score [%] and in vascular volume [%] on HRCT at week 26. Absolute change from baseline in forced vital capacity (FVC) [mL] at week 26 is a secondary outcome, unlike the FIBRONEER-ILD trial. The study is scheduled to end in 2027.

## 5. Conclusions

Systemic autoimmune rheumatic disease-associated interstitial lung disease represents a complex therapeutic landscape in which inflammatory and fibrotic mechanisms coexist and frequently drive progressive pulmonary fibrosis despite conventional immunosuppressive therapy. The emergence of antifibrotic strategies has reshaped disease management, yet significant unmet needs remain, particularly with regard to treatment tolerability, long-term adherence, and integration with background immunomodulatory regimens.

Preferential PDE4B inhibition with nerandomilast introduces a mechanistically distinct approach that targets intracellular signaling pathways implicated in both inflammation and fibrogenesis. Evidence derived from progressive pulmonary fibrosis trials demonstrates a consistent attenuation of lung function decline in the overall population, while subgroup analyses suggest a potential benefit in autoimmune-associated ILD, although estimates remain limited by statistical imprecision and trial designs not specifically tailored to SARD-ILD populations. Importantly, the safety profile observed across early clinical development programs indicates a tolerability pattern that differs from existing antifibrotic therapies, characterized by predominantly gastrointestinal adverse events and the need for continued evaluation of neuropsychiatric risk in broader real-world settings.

Interpretation of current data in SARD-ILD requires careful consideration of protocol-driven restrictions on immunosuppressive therapy, which may limit external validity and complicate extrapolation to routine clinical practice where combination strategies are common. The relatively low exposure to key immunomodulatory agents within clinical trials underscores the need for dedicated studies designed to evaluate nerandomilast in conjunction with optimized systemic therapy.

## Figures and Tables

**Table 1:** Main trials for antifibrotics use in SARD-ILD. * Specific data for SARD-ILD population have not been published.

	Nintedanib	Pirfenidone	Nerandomilast
**Mechanism of action**	Inhibitor of the receptor tyrosine kinases PDGFR, FGFR and VEGFR and non-receptor tyrosine kinases of the Src family	Inhibition of TGF-b-induced signaling pathways	Preferential inhibitor of PDE4B
**Efficacy**	INBUILD—[10] [PPF]: slows the annual decline of FVC by 104 mL per year compared with placebo SENSCIS—[5] [SSc-ILD]: slows the annual decline of FVC by 41 mL per year compared with placebo	RELIEF *—[11] PPF: early stopped for interim analysis—lower decline in FVC % predicted in the pirfenidone versus placebo TRAIL1—[12] [RA-ILD]: early stopped for COVID-19 pandemic and slow recruitment. Pirfenidone reduces the annual decline of FVC by 80.8 mL per year compared with placebo in patients with RA-ILD SCLERODERMA LUNG STUDY III—[13] [SSc-ILD]: no significant advantages in FVC% in adding pirfenidone to MMF during 18 month-follow-up PIRFENIVAS—[14] [AAV-ILD]: in a phase II pilot study pirfenidone increased FVC at 52 weeks compared to baseline (+7.2% predicted; +192 mL)	FIBRONEER-ILD—[15] [PPF] improves FVC at week 52 by 45.9 mL (9 mg BID) and 42.2 mL (18 mg BID) vs. placebo
**Dose**	150 mg twice daily	801 mg tablets three times a day	18 mg twice daily or 9 mg twice daily
**Common side effects**	Diarrhea (63–75.7%), weight loss (11–12%), elevated liver enzymes (5–15%)	Nausea (50%), diarrhea (31–55%), photosensitivity reaction (10–14%) Elevated liver enzymes (<5%)	Diarrhea [36.6% (18 mg BID)–29.5% (9 mg BID)], weight loss [10.7% (18 mg BID)–6.9% (9 mg BID)], nasopharyngitis [8.7% (18 mg BID)–11.5% (9 mg BID)]
**Enzyme metabolism**	Esterase cleavage (major), CYP3A4 (minor)	CYP1A2	CYP3A4
**Cautions**	Risk of bleeding or gastrointestinal perforation; VKA and dual antiplatelet therapy should be considered with caution	Drug interactions with CYP1A2 inhibitors or inducers	Depression or suicidal behavior
**Need for blood test monitoring**	Yes	Yes	No

Abbreviation: PDGFR: Platelet-Derived Growth Factor Receptor; FGFR: Fibroblast Growth Factor Receptor; VEGFR: Vascular Endothelial Growth Factor Receptor; PDE4B: phosphodiesterase 4B; VKA: vitamin K antagonist. * extrapolated data from SARD-ILD not available.

## Data Availability

All data is contained within the article.
